# Supplementary material for: Sequential Hypofractionated versus Concurrent Twice-Daily Radiotherapy for Limited-Stage Small-Cell Lung Cancer: A Propensity Score-Matched Analysis
Source: Cancers (Basel). 2022 Aug 13;14(16):3920. doi: 10.3390/cancers14163920 (PMC9406024; doi:10.3390/cancers14163920)
Supplement: Supplementary file 1 [file cancers-14-03920-s001.zip › cancers-1823904-supplementary.pdf]

Supplemental Table S1. Univariable Cox analysis of OS, RFS and DMFS Before PSM.

Variables	OS		RFS		DMFS	
	HR(95%CI)	*P*	HR(95%CI)	*P*	HR(95%CI)	*P*
**Age (year)**	0.992(0.966-1.019)	0.578	0.995(0.970-1.021)	0.714	1.003(0.976-1.030)	0.853
**SER (day)**	1.001(0.997-1.004)	0.771	1.000(0.997-1.004)	0.813	0.999(0.995-1.002)	0.474
**Total chemo cycles (times)**	1.120(0.946-1.326)	0.187	1.137(0.966-1.339)	0.123	1.032(0.881-1.208)	0.698
**Chemo cycles before radiotherapy (times)**	1.130(0.953-1.340)	0.161	1.129(0.960-1.328)	0.144	1.021(0.869-1.199)	0.803
**Total radiotherapy time (day)**	1.033(0.992-1.076)	0.116	1.029(0.989-1.071)	0.160	1.021(0.977-1.066)	0.353
**Group**						
**Twice daily**	1.000		1.000		1.000	
**Sequential-hypo**	0.502(0.235-1.074)	0.076	0.572(0.287-1.140)	0.112	0.546(0.275-1.084)	0.084
**Age (year)**						
**<50**	1.000		1.000		1.000	
**≥50**	0.640(0.357-1.147)	0.134	0.727(0.410-1.290)	0.276	0.702(0.401-1.229)	0.216
**Sex**						
**Male**	1.000		1.000		1.000	
**Female**	0.967(0.513-1.823)	0.918	0.937(0.509-1.725)	0.835	0.795(0.434-1.457)	0.458

**ECOG PS**						
**0-1**	1.000		1.000		1.000	
**2-3**	O.940(0.440-2.009)	0.874	0.853(0.266-2.740)	0.790	1.049(0.379-2.900)	0.927
**Smoking**						
**Never smoker**	1.000		1.000		1.000	
**Former smoker**	2.166(0.903-5.196)	0.084	2.368(1.000-5.612)	**0.050**	1.242(0.590-2.615)	0.568
**Current smoker**	2.222(0.960-5.143)	0.062	2.366(1.029-5.437)	**0.043**	1.307(0.646-2.643)	0.457
**PCI**						
**no**	1.000		1.000		1.000	
**yes**	1.034(0.541-1.976)	0.919	1.055(0.566-1.966)	0.866	0.755(0.424-1.347)	0.342
**Introduction chem**						
**no**	1.000		1.000		1.000	
**yes**	22.864(0.243-2150.682)	0.177	4.766(0.658-34.493)	0.122	4.760(0.658-34.428)	0.122
**SER (day)**						
**<90**	1.000		1.000		1.000	
**≥90**	1.504(0.807-2.801)	0.199	1.410(0.784-2.536)	0.251	1.105(0.629-1.942)	0.728
**Total radiotherapy time (day)**						

**<24**	1.000		1.000		1.000	
**≥24**	1.934(1.072-3.488)	**0.028**	1.857(1.051-3.280)	**0.033**	1.449(0.817-2.570)	0.205
**Total chemo cycles (times)**						
**<4**	1.000		1.000		1.000	
**≥4**	1.549(0.824-2.909)	0.174	1.551(0.845-2.846)	0.157	1.161(0.658-2.050)	0.606
**Chemo cycles before radiotherapy (times)**						
**<2**	1.000		1.000		1.000	
**≥2**	2.043(0.736-5.674)	0.170	1.808(0.721-4.536)	0.207	1.464(0.628-3.415)	0.377
**T stage**						
**1-2**	1.000		1.000		1.000	
**3-4**	1.203(0.600-2.412)	0.603	1.390(0.730-2.649)	0.317	0.934(0.471-1.853)	0.846
**N stage**						
**0-1**	1.000		1.000		1.000	
**2-3**	2.178(0.928-5.110)	0.074	2.472(1.058-5.776)	**0.037**	2.742(1.175-6.400)	**0.020**
**AJCC stage**						
**I- II**	1.000		1.000		1.000	
**III**	2.158(0.920-5.062)	0.077	2.374(1.016-5.550)	**0.046**	2.696(1.155-6.289)	**0.022**

**Comorbidity**						
**No**	1.000		1.000		1.000	
**Yes**	1.486(0.810-2.723)	0.201	1.259(0.649-2.282)	0.449	1.454(0.819-2.581)	0.201

Hazard ratios and 95% confidence intervals were calculated by a stratified Cox proportional hazards model. Abbreviations: OS, overall survival; RFS, Recurrence-free survival; DMFS, Distant Metastasis-free Survival; ECOG PS: Eastern Cooperative Oncology Group Performance Status; PCI, Prophyctic Cranial Irradiation; SER, from the date of Start treatment to the End Radiotherapy ; P values less than 0.05 are highlighted in bold.

Supplemental Table S2. Mutivariable Cox analysis of OS, RFS and DMFS Before PSM.

**Variables**	**OS**		**RFS**		**DMFS**	
	**HR(95%CI)**	***P***	**HR(95%CI)**	***P***	**HR(95%CI)**	*P*
**Group**						
**Twice daily**	1.000				1.000	
**sequential-Hypo**	0.353(0.161-0.775)	0.009			0.483(0.242-0.962)	0.039
**Total radiotherapy time (day)**						
**<24**	1.000		1.000			
**≥24**	2.452(1.335-4.503)	0.004	1.774(1.004-3.132)	0.048		
**AJCC stage**						
**I- II**	1.000					

**III**	2.310(0.982-5.432)	0.055				
**T stage**						
**1-2**						
**3-4**						
**N stage**						
**0-1**			1.000		1.000	
**2-3**			2.369(1.013-5.543)	0.047	3.032(1.295-7.103)	0.011

Hazard ratios and 95% confidence intervals were calculated by a stratified Cox proportional hazards model. Abbreviations: OS, overall survival; RFS, Recurrence-free survival; DMFS, Distant Metastasis-free Survival; PS: Performance Status; P values less than 0.05 are highlighted in bold.

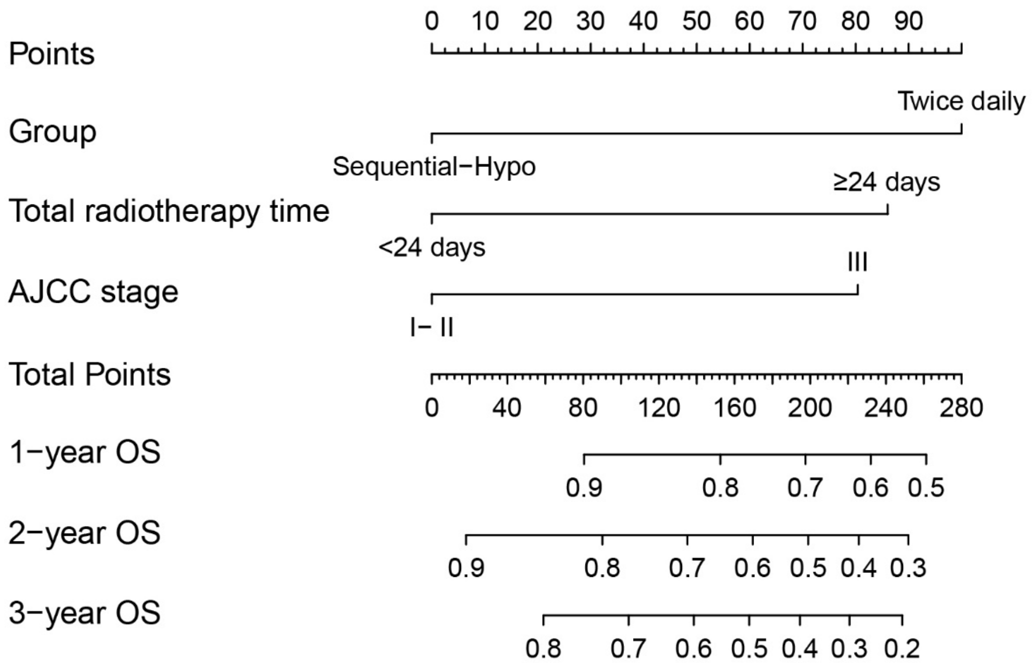

Figure S1. The nomogram of OS before PSM.

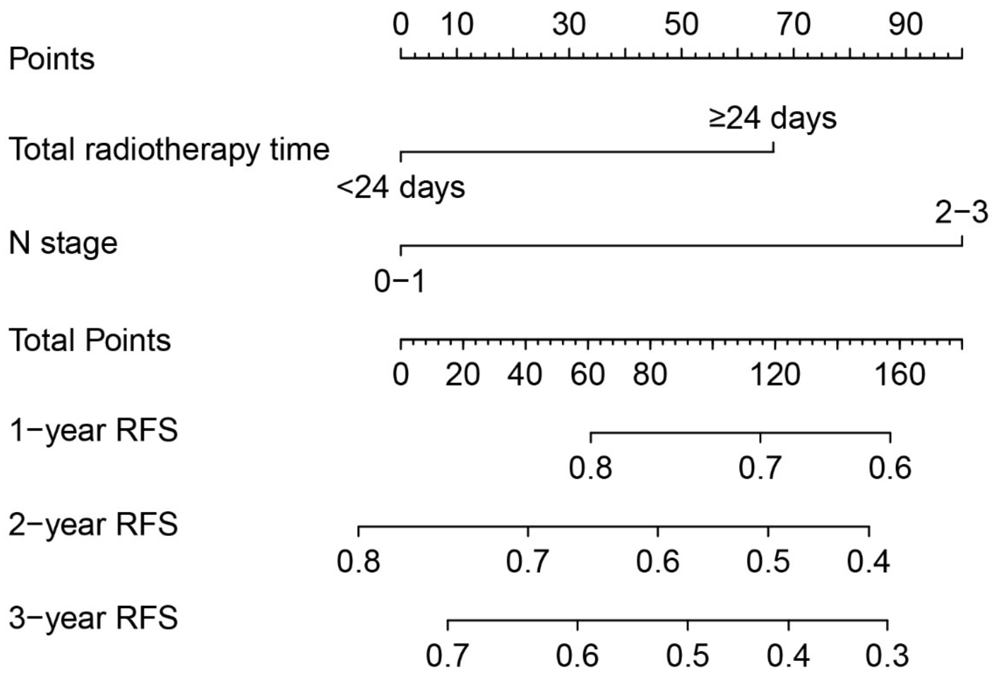

Figure S2. The nomogram of RFS before PSM.

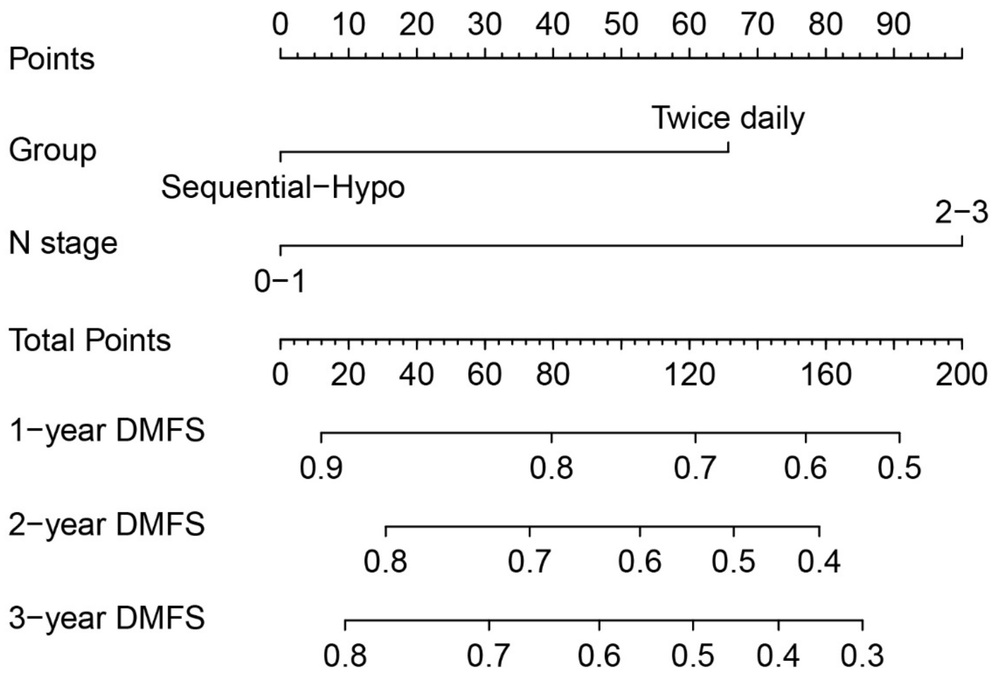

Figure S3. The nomogram of DMFS before PSM.

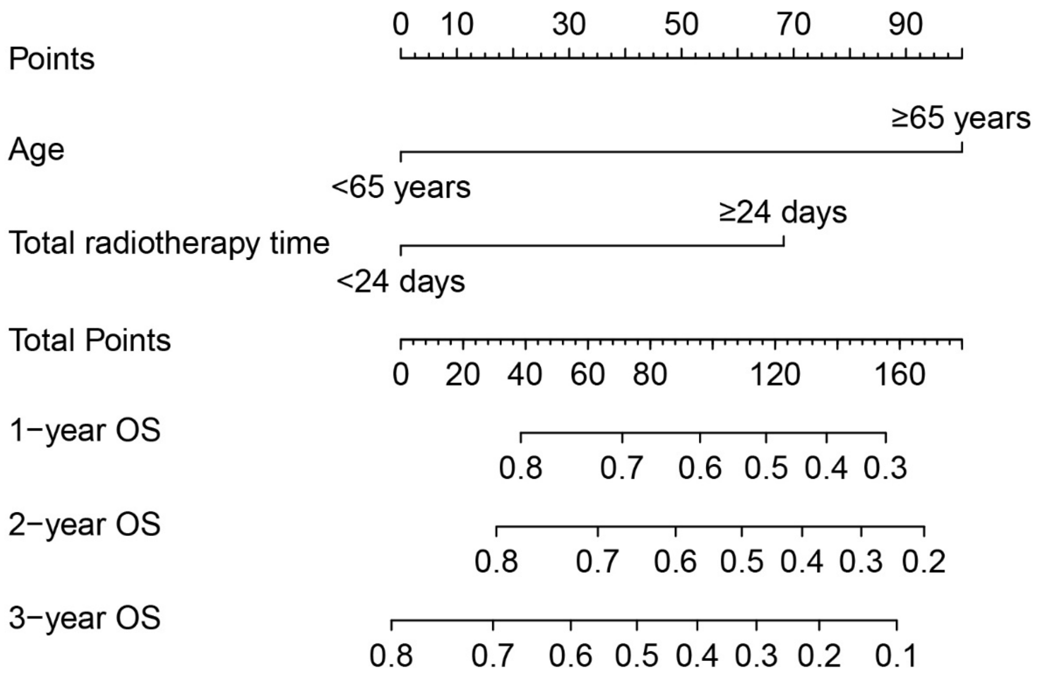

Figure S4. The nomogram of OS after PSM.

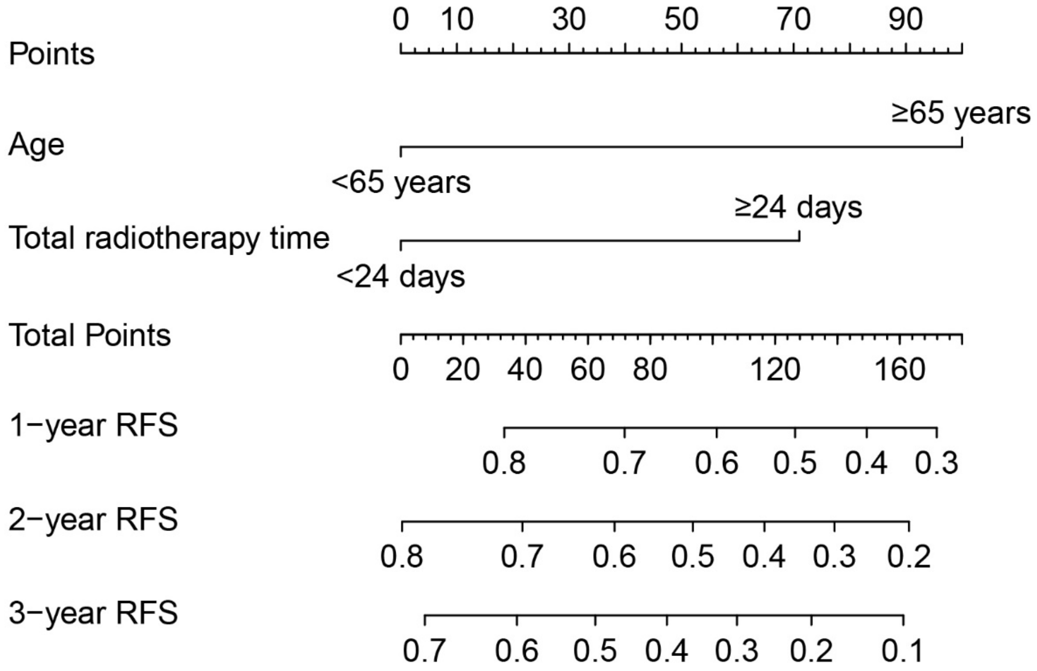

Figure S5. The nomogram of RFS after PSM.
